# Necrolytic acral erythema in an incarcerated population: A case series

**DOI:** 10.1016/j.jdcr.2025.12.038

**Published:** 2025-12-29

**Authors:** Iman Ali, Mary Cavanagh, Brent C. Kelly

**Affiliations:** a Department of Internal Medicine, Texas Health Presbyterian Dallas, Dallas, Texas; b Department of Dermatology, University of Texas Medical Branch Medical School, Galveston, Texas

**Keywords:** hepatitis C, incarcerated, necrolytic acral erythema, nutritional deficiency, zinc

## Introduction

Necrolytic acral erythema (NAE) is a rare dermatological condition predominantly associated with chronic hepatitis C virus (HCV) infection and zinc deficiency.^1^^,^^2^ Clinically, this condition presents as well-demarcated, hyperkeratotic, scaly plaques with secondary lichenification and hyperpigmentation commonly involving acral surfaces.^1^

Although the pathogenesis of NAE is poorly understood, it is thought to involve a complex interaction between hypozincemia and chronic liver disease, particularly HCV.^3^^,^^4^ Cases of NAE-like eruptions have been reported in seronegative individuals with hypozincemia secondary to Crohn’s disease, Celiac disease, and gastric bypass surgery.^5^^,^^6^^,^^7^ These individuals improved with zinc supplementation, further emphasizing the critical role of zinc in NAE development.

Diagnosis of NAE is primarily clinical, supported by histological features such as epidermal necrosis, psoriasiform hyperplasia, and superficial perivascular inflammation, though these findings are supportive, not diagnostic.^1^ Management includes oral zinc replacement, treatment of underlying liver disease, and topical anti-inflammatory agents, such as corticosteroids and calcineurin inhibitors.^8^ Treatment responses can vary, further supporting a multifactorial pathogenesis.^9^

HCV prevalence is significantly higher in incarcerated populations. In the United States, an estimated 15.2% of inmates are HCV-seropositive (ranging from 8.3% in Nebraska to 38.8% in Alaska). Of those who are seropositive, 8.7% are viremic and 54.9% have detectable HCV RNA.^10^ This represents a nearly 9-fold higher prevalence of HCV in state prisons compared to the general US population. Globally, HCV prevalence is estimated at 2.5% while rates among incarcerated individuals are estimated at 17.7%, with the highest rates reported in Australia and Oceania (28.4%).^11^^,^^12^ Based on these findings, cutaneous manifestations of HCV may be disproportionately represented in incarcerated populations.

This series describes 7 incarcerated patients with HCV and zinc deficiency who developed NAE and demonstrated significant improvement with zinc therapy.

## Case series:

### Case number 1

**Fig 1:**
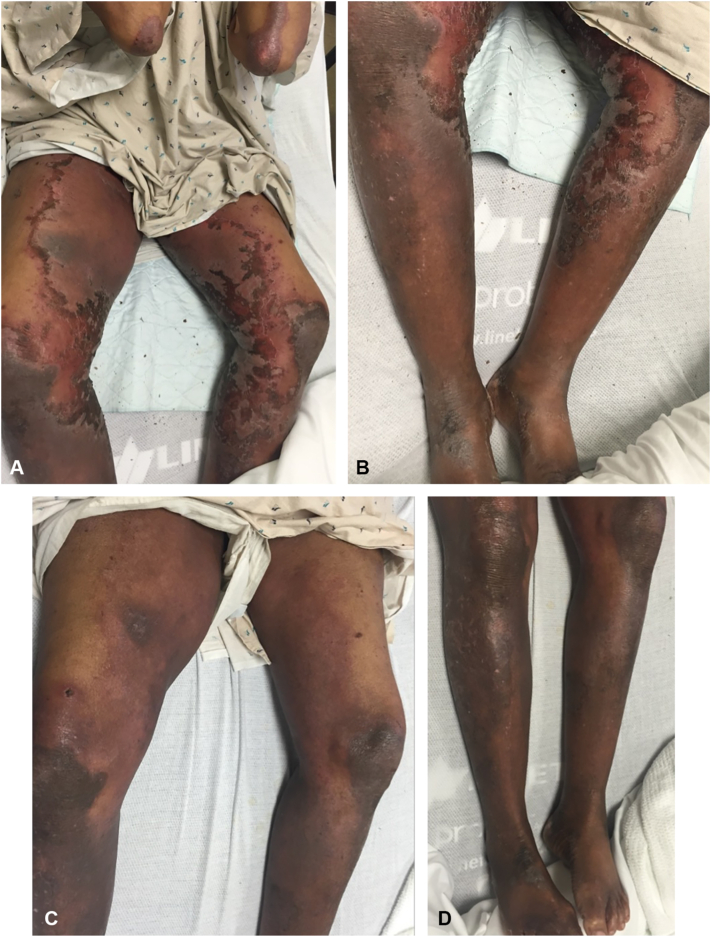
**A,** and **B,** Violaceous erythematous scaly annular plaques and hyperpigmented scaly patches on the bilateral lower extremities before zinc therapy **C,** and **D,** Improvement of NAE after 8 months of zinc supplementation. *NAE*, Necrolytic acral erythema.

A 60-year-old man with a history of squamous cell carcinoma of the penis, Bowenoid papulosis, HCV genotype 1a, and hepatocellular carcinoma (HCC) treated with radiofrequency ablation x2 and transarterial chemoembolization (TACE) presented with a long-standing history of tender, pruritic hyperpigmented lichenified patches on the elbows and hyperpigmented scaly patches on the lower extremities (Fig 1, *A* and *B*). Prior use of several topical corticosteroids and emollients was ineffective, and previous biopsies were nonspecific, with a differential including lichen planus, lichen simplex chronicus, and psoriasiform dermatitis.

Laboratory results revealed low serum zinc at 56 μg/dL (normal: 60-120 μg/dL). Zinc supplementation was initiated with oral zinc sulfate 1100 mg daily, and adjunctive betamethasone ointment. During his initial work-up for HCV, his HCC was discovered. Therefore, he did not begin antiviral therapy. At subsequent follow-up 8 months later, he demonstrated marked clinical improvement with zinc levels rising to 70 μg/dL (Fig 1, *C* and *D*). Symptoms briefly recurred after a 3-day lapse in supplementation and resolved upon resumption. He passed away a year after his follow-up appointment from a myocardial infarction.

### Case number 2

**Fig 2:**
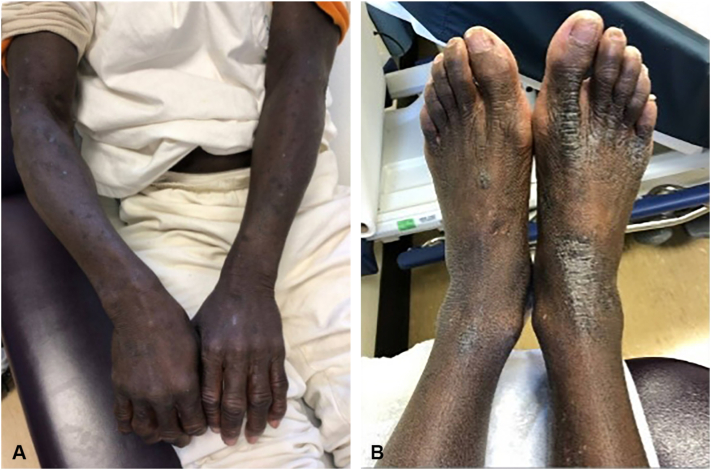
**A,** Diffuse scale with hyperpigmented lichenoid plaques on bilateral arms and hands before zinc therapy. **B,** Diffuse scale with hyperpigmented lichenoid plaques on bilateral feet before zinc therapy.

A 58-year-old man with HCV genotype 1a presented with a 2-year history of pruritic hyperpigmented lichenified plaques on the hands (Fig 2, *A*), feet (Fig 2, *B*), arms, and trunk. Laboratory evaluation showed low serum zinc at 38 μg/dL with normal liver enzymes. He was treated with sofosbuvir/velpatasvir but subsequently experienced virologic relapse. He was diagnosed with NAE and started on oral zinc sulfate 220 mg 4 times daily with adjunctive triamcinolone 0.1% cream while receiving sofosbuvir/velpatasvir. At follow-up, the patient reported marked cutaneous improvement and complete resolution of pruritus, which preceded documented virologic relapse. A few years later, he was successfully treated with a 12-week course of glecaprevir/pibrentasvir. One year after treatment initiation, he experienced a NAE flare following discontinuation of zinc.

### Case number 3

**Fig 3:**
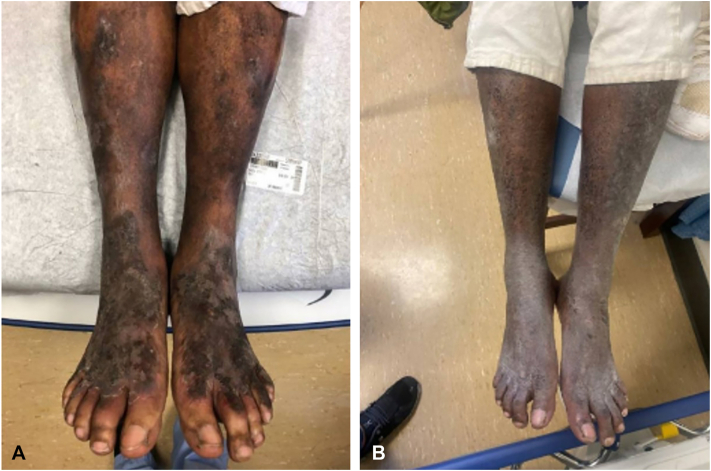
**A,** Violaceous erythematous scaly annular plaques on the bilateral lower extremities before zinc therapy. **B,** Visible improvement of NAE after a year and a half on zinc supplements. *NAE*, Necrolytic acral erythema.

A 56-year-old man with HCV genotype 4 treated with sofosbuvir/velpatasvir and ribavirin, HCC status post TACE with no recurrence seen on computed tomography 6 years later, HIV (on antiretroviral therapy), and insulin-dependent diabetes mellitus presented with pruritic, scaly plaques on the trunk and extremities that had persisted over 10 years. Prior treatments including topical steroids, prednisone, oral antibiotics, and topical emollients were ineffective, and prior skin biopsy demonstrated psoriasiform dermatitis. Continued therapy with triamcinolone 0.01% twice daily and light therapy moderately improved his symptoms, with progression on the lower extremities and anterior tibial areas (Fig 3, *A*).

A serum zinc level was later found to be profoundly low at 36 μg/dL, supporting a diagnosis of NAE. Zinc sulfate 150 mg/day was initiated, and he continued fluocinonide 0.05% cream twice daily. At subsequent follow-up a year and a half later, the patient reported significant improvement (Fig 3, *B*).

### Case number 4

**Fig 4:**
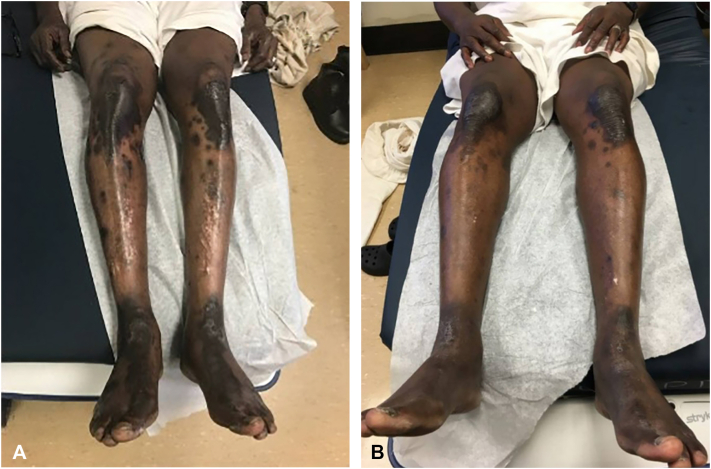
**A,** Excoriated and lichenified hyperpigmented papules with postinflammatory changes on the bilateral legs before zinc therapy. **B,** Improvement of NAE on the bilateral legs after 1 year of oral zinc supplement. *NAE*, Necrolytic acral erythema.

A 53-year-old man with type 2 diabetes and HCV genotype 1a presented with excoriated and lichenified hyperpigmented plaques on his bilateral lower extremities which had been present for years (Fig 4, *A*), causing significant pruritus and sleep disturbance. Prior biopsies showed prurigo nodularis and lichen simplex chronicus. Previous failed treatments included a 10-day course of oral prednisone (20 mg daily), antihistamines, multiple topical corticosteroids, and emollients.

Laboratory results revealed low serum zinc level at 50 μg/dL and mildly elevated liver enzymes (ALT 66 U/L, AST 73 U/L; normal ALT and AST: 0-35 U/L). Zinc supplementation was initiated with zinc sulfate 440 mg daily, along with triamcinolone 0.1% cream as needed. At 2-month follow-up, he demonstrated both clinical improvement (Fig 4, *B*) and an increase in zinc level to 64.4 μg/dL.

Of note, this improvement was seen prior to the initiation of antiviral therapy. He subsequently received sofosbuvir/velpatasvir; however, HCV RNA remained detectable 4 weeks post-treatment. He was then treated with sofosbuvir–velpatasvir–voxilaprevir for 12 weeks.

### Case number 5

**Fig 5:**
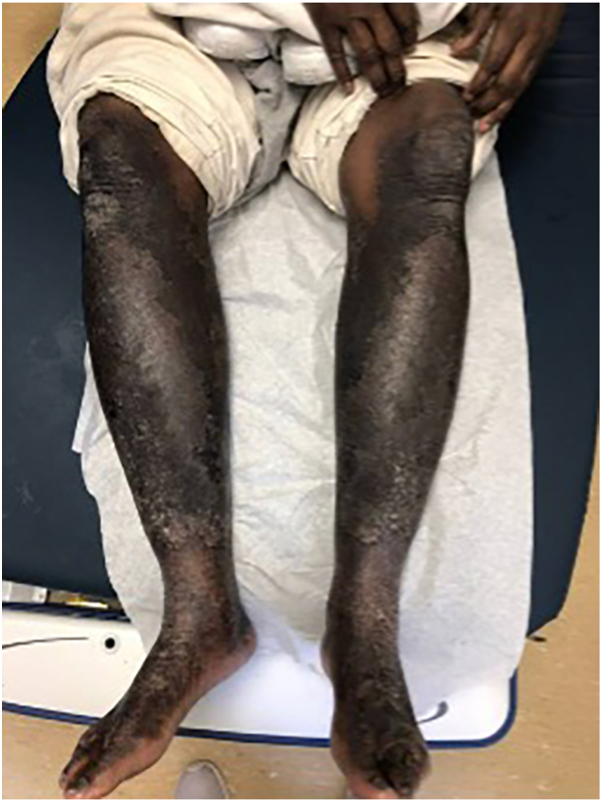
Hyperpigmented lichenified plaques present on the bilateral lower legs present before zinc treatment

A 59-year-old man with chronic HCV genotype 1a and HCC status post TACE presented with a 5-year history of pruritic hyperpigmented lichenified plaques beginning on his feet and spreading to the lower legs (Fig 5), arms, and chest. He had been previously diagnosed with psoriasis and a concurrent diagnosis of tinea corporis. He had failed treatment with topical emollients, clotrimazole, and triamcinolone.

Laboratory studies revealed a low zinc level of 26.8 μg/dL, HCV viral load of 1,893,616 IU/mL by nucleic acid amplification test, and elevated liver enzymes (ALT 163 U/L, AST 267 U/L, and alkaline phosphate 134 U/L). Punch biopsy showed mild acanthosis without psoriasiform changes and focal epidermal pallor but was otherwise nonspecific. Given the clinical findings, hypozincemia, and HCV status, a diagnosis of NAE was made.

Zinc sulfate was initiated at 3 mg/kg daily with fluocinonide 0.05% ointment twice daily, resulting in clinical improvement within 3 months. He noted 1 recurrence when he briefly discontinued zinc. The patient declined antiviral therapy for HCV and was released from custody without receiving treatment.

### Case number 6

**Fig 6:**
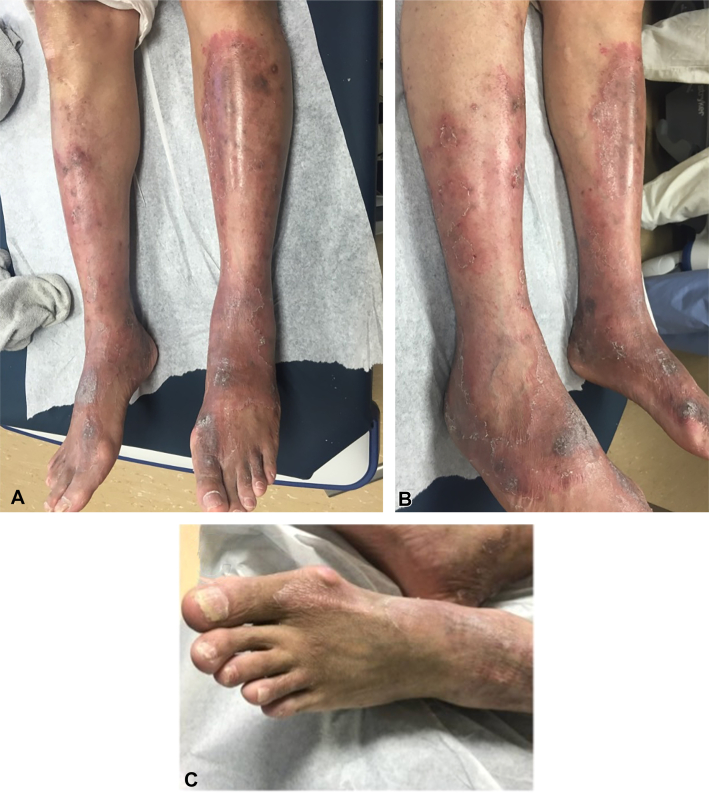
**A,** and **B,***Red-brown mottled* plaques with “peeling paint” scale on the bilateral legs along with lichenified plaques on the dorsal feet before zinc treatment. **C,** Bright erythematous borders and few lichenified areas on the dorsal feet after zinc supplementation.

A 54-year-old male with rheumatoid arthritis (RA) and HCV genotype 4 presented with a 2-year history of pruritic red-brown mottled plaques with “peeling paint” scale on the legs along and lichenified plaques on the dorsal feet (Fig 6, *A* and *B*), along with genital pruritus and scale. His medications included hydroxychloroquine and etanercept for RA. He had tried topical clotrimazole and oral fluconazole without improvement.

The patient developed RA while receiving pegylated interferon for HCV, with elevated anticyclic citrullinated peptide antibody levels, leading to discontinuation of antiviral therapy. He was released from custody 6 years later without further HCV-directed treatment.

A previous skin biopsy revealed spongiotic dermatitis with hyperkeratosis and acanthosis consistent with lichen simplex chronicus. Laboratory results showed low serum zinc (55 μg/dL), mildly elevated ALT (53 U/L), and normal AST (31 U/L). Zinc supplementation was initiated; however, he could not tolerate the 220 mg dose due to nausea. A reduced dose was tolerated but ineffective, and after 4 months there was no clinical improvement (Fig 6, *C*), with zinc levels remaining low at 56 μg/dL. Although NAE was suspected, the patient was lost to follow-up before adequate zinc repletion could be achieved, and treatment response remains unknown.

### Case number 7

**Fig 7:**
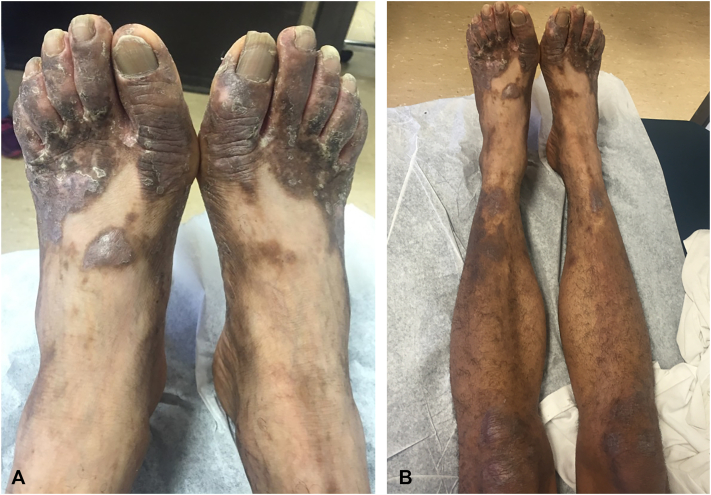
**A,** Lichenified hyperpigmented scaly papules coalescing into plaques on the dorsal feet before zinc therapy. **B,** Lichenified hyperpigmented scaly papules coalescing into plaques on the dorsal feet and involving the bilateral lower extremities before zinc therapy.

A 60-year-old male with HCV genotype 1a and a history of ischemic stroke presented with a 3-year history of lichenified hyperpigmented scaly papules coalescing into plaques on the dorsal feet and bilateral lower extremities (Fig 7, *A* and *B*), which began at the ankles and gradually spread to his entire body. He reported generalized pruritus, most severe on the dorsal feet. He had tried hydrocortisone cream and petrolatum without any improvement.

Laboratory studies revealed low serum zinc level at 59 μg/dL with normal liver enzymes (ALT of 50 U/L, AST of 43 U/L, and alkaline phosphate of 52 U/L) and he was initiated on zinc supplementation. He began antiviral therapy with sofosbuvir/velpatasvir 5 years later but passed away shortly after; therefore, treatment response could not be assessed. Given hypozincemia in the setting of chronic HCV infection, his presentation was most consistent with NAE.

## Discussion

NAE is a rare cutaneous manifestation of HCV influenced by zinc deficiency, chronic liver disease, altered nutrient absorption, gut microbiome disruption, and systemic inflammation. Chronic hepatic inflammation in HCV alters zinc metabolism, redirecting zinc toward hepatic enzymatic activity and reducing its availability to peripheral tissues. Zinc is an important cofactor for various enzymes involved in DNA repair, antioxidant defense, epithelial integrity, and immune regulation therefore deficiency predisposes to cutaneous breakdown and inflammation.^13^

Disruption of the gut microbiome in chronic liver disease and HCV infection further reduces zinc absorption through decreased transporter expression, increased intestinal permeability, and inflammatory changes.^14^ This theory of disruption in the gut microbiome aligns with similar findings in other zinc deficient conditions such as Celiac and Crohn’s disease. Proinflammatory cytokines such as TNF-ɑ and IL-6, elevated in HCV infection, may redistribute zinc systemically and promote cutaneous inflammation thereby exacerbating skin manifestations such as those seen in NAE.^14^

Dietary intake of zinc in the incarcerated population is unknown, but it could be a contributing factor to zinc deficiency. The high prevalence of HCV within prisons amplifies the likelihood of encountering NAE in this population. Consistent with prior reports, most patients in this series improved with zinc supplementation. Notably, several patients experienced recurrence of symptoms upon interruption or discontinuation of zinc therapy, further supporting the central role of zinc deficiency in disease pathogenesis.

Several zinc formulations exist, including sulfate, gluconate, acetate, citrate, and oxide. Zinc sulfate is the most widely used form in NAE treatment, most likely due to its higher elemental zinc content and widespread availability. The standard regimen for NAE treatment is 220 mg twice daily for up to 8 weeks. However, dosing in this series varied based on deficiency severity, patient weight, gastrointestinal tolerance, and physician preference. Higher or weight-based dosing was used in patients with profound deficiency, while lower doses were selected when nausea limited adherence. Common adverse effects include nausea (as seen in Case 6), vomiting, diarrhea, abdominal pain, headaches, and dizziness.

Although Cases 6 and 7 lacked documented improvement, they highlight challenges in the management of NAE, including intolerance to zinc, difficulty maintaining follow-up in incarcerated populations, and long timelines (months to over a year) required to assess treatment response, which can be difficult in both prison and community populations.

The Texas Department of Criminal Justice, in partnership with The University of Texas Medical Branch through the Correctional Managed Health Care Committee, has maintained a structured HCV management program since the early 1990s. All incarcerated individuals are screened for HCV upon intake, and those who test positive are enrolled in the chronic virology clinic for ongoing evaluation. Treatment eligibility is determined using multiple clinical factors, including APRI score, to triage patients for direct-acting antiviral therapy. While current management relies on newer available direct-acting antivirals, earlier management included interferon and ribavirin, which were the only therapeutic options and were limited to select viral phenotypes, restricting access for many patients. This historical context explains why several individuals in this series were evaluated during periods when antiviral therapy was unavailable or difficult to obtain.

Importantly, improvement in cutaneous disease across this series often correlated more closely with zinc repletion than with HCV treatment status. Several patients demonstrated clinical improvement prior to antiviral therapy initiation or in the absence of virologic cure, supporting zinc deficiency as a primary driver of NAE independent of viral clearance. These findings emphasize the importance of recognizing and treating zinc deficiency even when HCV therapy is delayed or incomplete.

Future studies should focus on elucidating the exact pathogenesis of NAE and further characterize factors affecting zinc absorption particularly given the overlap with other malabsorptive conditions, such as Crohn’s and Celiac disease. Improved recognition of HCV-associated dermatologic disease is especially important in incarcerated populations, where targeted diagnosis and management can substantially improve patient outcomes.

## Conflicts of interest

None disclosed.
